# Supplementary material for: Novel Driver Strength Index highlights important cancer genes in TCGA PanCanAtlas patients
Source: PeerJ. 2022 Aug 11;10:e13860. doi: 10.7717/peerj.13860 (PMC9375969; doi:10.7717/peerj.13860)
Supplement: Supplemental Information 2 [file peerj-10-13860-s002.zip › COHORTS/genes plots/detailed/2021_8_16_14_9_distribution_events_detailed_females_27.pdf]

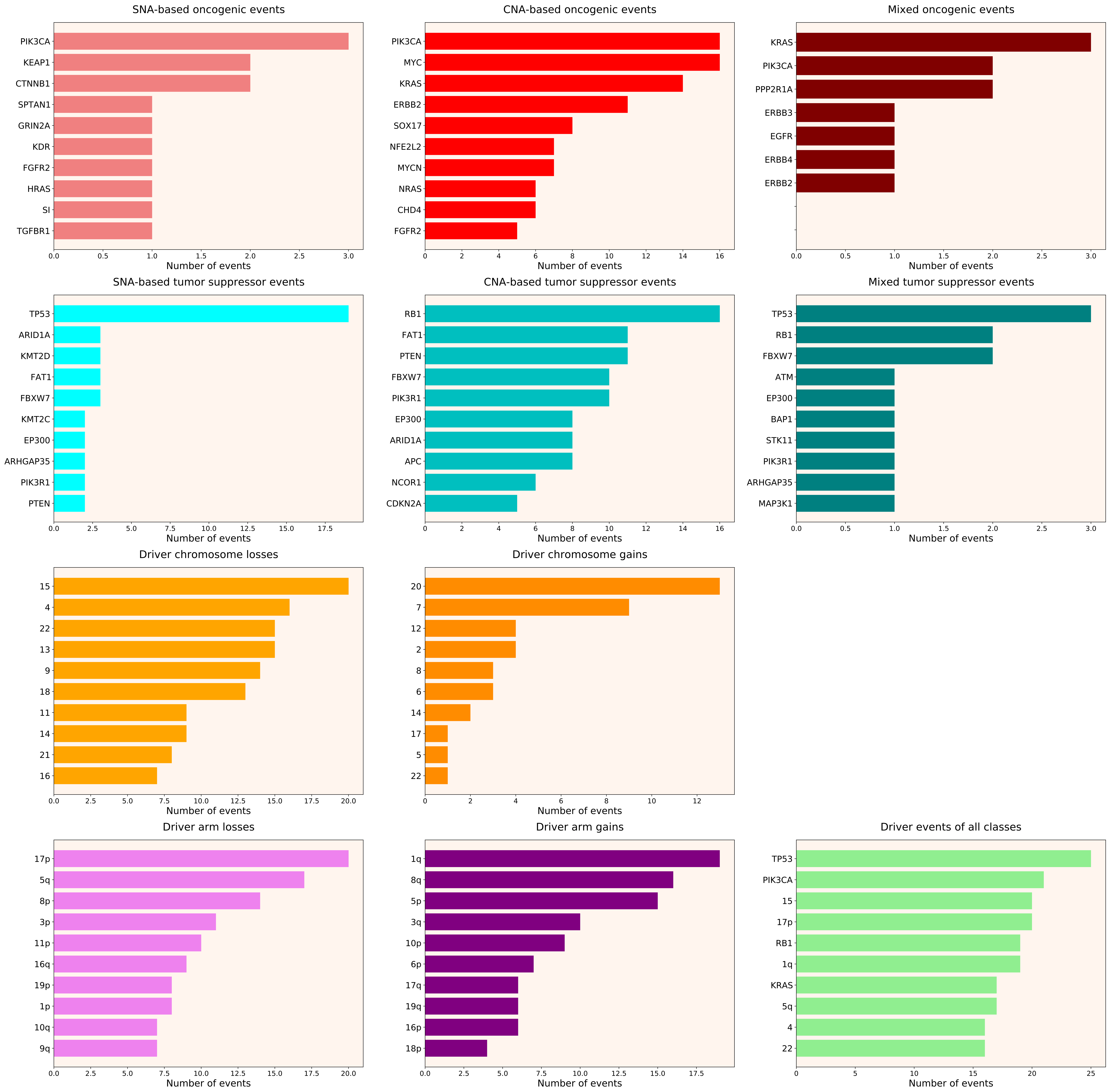
SNA-based oncogenic events
PIK3CA
KEAP1
CTNNB1
SPTAN1
GRIN2A
KDR
FGFR2
HRAS
SI
TGFBR1
0.0 0.5 1.0 1.5 2.0 2.5 3.0
Number of events
CNA-based oncogenic events
PIK3CA
MYC
KRAS
ERBB2
SOX17
NFE2L2
MYCN
NRAS
CHD4
FGFR2
0 2 4 6 8 10 12 14 16
Number of events
Mixed oncogenic events
KRAS
PIK3CA
PPP2R1A
ERBB3
EGFR
ERBB4
ERBB2
0.0 0.5 1.0 1.5 2.0 2.5 3.0
Number of events
SNA-based tumor suppressor events
TP53
ARID1A
KMT2D
FAT1
FBXW7
KMT2C
EP300
ARHGAP35
PIK3R1
PTEN
0.0 2.5 5.0 7.5 10.0 12.5 15.0 17.5
Number of events
CNA-based tumor suppressor events
RB1
FAT1
PTEN
FBXW7
PIK3R1
EP300
ARID1A
APC
NCOR1
CDKN2A
0 2 4 6 8 10 12 14 16
Number of events
Mixed tumor suppressor events
TP53
RB1
FBXW7
ATM
EP300
BAP1
STK11
PIK3R1
ARHGAP35
MAP3K1
0.0 0.5 1.0 1.5 2.0 2.5 3.0
Number of events
Driver chromosome losses
15
4
22
13
9
18
11
14
21
16
0.0 2.5 5.0 7.5 10.0 12.5 15.0 17.5 20.0
Number of events
Driver chromosome gains
20
7
12
2
8
6
14
17
5
22
0 2 4 6 8 10 12
Number of events
Driver arm losses
17p
5q
8p
3p
11p
16q
19p
1p
10q
9q
0.0 2.5 5.0 7.5 10.0 12.5 15.0 17.5 20.0
Number of events
Driver arm gains
1q
8q
5p
3q
10p
6p
17q
19q
16p
18p
0.0 2.5 5.0 7.5 10.0 12.5 15.0 17.5
Number of events
Driver events of all classes
TP53
PIK3CA
15
17p
RB1
1q
KRAS
5q
4
22
0 5 10 15 20 25
Number of events
